# p53 Affects Zeb1 Interactome of Breast Cancer Stem Cells

**DOI:** 10.3390/ijms24129806

**Published:** 2023-06-06

**Authors:** Sergey E. Parfenyev, Sergey V. Shabelnikov, Elena N. Tolkunova, Nickolai A. Barlev, Alexey G. Mittenberg

**Affiliations:** 1 Institute of Cytology of the Russian Academy of Sciences, St. Petersburg 194064, Russia; gen21eration@gmail.com (S.E.P.); buddasvami@gmail.com (S.V.S.); entolk62@mail.ru (E.N.T.); 2 Department of Biomedical Sciences, School of Medicine, Nazarbayev University, Astana 20000, Kazakhstan

**Keywords:** breast cancer, cancer stem cells, epithelial-to-mesenchymal transition (EMT), metastasis, p53, Zeb1

## Abstract

P53 is a critical tumor suppressor that protects the integrity of genome and prevents cells from malignant transformation, including metastases. One of the driving forces behind the onset of metastases is the epithelial to mesenchymal transition (EMT) program. Zeb1 is one of the key transcription factors that govern EMT (TF-EMT). Therefore, the interaction and mutual influence of p53 and Zeb1 plays a critical role in carcinogenesis. Another important feature of tumors is their heterogeneity mediated by the presence of so-called cancer stem cells (CSCs). To this end, we have developed a novel fluorescent reporter-based approach to enrich the population of CSCs in MCF7 cells with inducible expression of Zeb1. Using these engineered cell lines, we studied the effect of p53 on Zeb1 interactomes isolated from both CSCs and regular cancer cells. By employing co-immunoprecipitations followed by mass spectrometry, we found that the composition of Zeb1 interactome was affected not only by the p53 status but also by the level of Oct4/Sox2 expression, indicating that stemness likely affects the specificity of Zeb1 interactions. This study, together with other proteomic studies of TF-EMT interactomes, provides a framework for future molecular analyses of biological functions of Zeb1 at all stages of oncogenesis.

## 1. Introduction

Breast cancer (BC) is a complex cancer disease, which is characterized by the high degree of recurrence after the initial surgical removal of the primary tumor followed by chemotherapy. One of the reasons behind this delayed reappearance of the tumor, often metastatic in nature, is the presence of remnant cancer stem cells that form micro-metastases in the surrounding tissues [1].

The epithelial-mesenchymal transition (EMT) is a key process for the transition of non-invasive cancer to invasive and increasing resistance to traditional chemotherapy, in which polarized epithelial cells lose their tight intercellular junctions, resulting in both increased migratory ability and invasive properties as well as the acquisition of a mesenchymal phenotype [2,3,4]. Understanding the functions of EMT markers associated with metastasis, as well as the mechanisms of their regulation, is critical for development of potential treatment strategies of patients with metastatic breast cancer, which is one of the deadliest types of BC in women worldwide with a mortality rate of over 2.1 per million cases per year [5].

Historically, the EMT program has been considered in terms of migration and invasion, however, in the last decade, it has been shown that EMT also induces the acquisition of stem properties by tumor cells [6]. In the case of carcinomas, these were defined as cancer stem cells [7,8]. Thus, for carcinoma of the breast, lung, pancreas, and colon origin, a relationship between EMT and acquisition of stem characteristics has been established [9]. Breast tumor CSCs have an increased tumor initiation potential and express various cell surface markers (e.g., low levels of CD24 and high levels of CD44) compared to other tumor cells [7,8]. Importantly, CSCs that are capable of becoming metastatic colony founders, are also highly resistant to anticancer treatment and immune response [6]. Therefore, the stemness status of cancer cells is clearly important for the survival of a cancer patient.

At present, only limited information is available about the mechanisms for acquiring stem properties during EMT activation. However, it is known that the main EMT factors (Snail, Twist or Zeb) are involved in this process. For example, Zeb1 has been shown to directly repress several miRNAs of the miR-200 family in pancreatic, breast, and lung cancer [10,11,12]. It was also shown that activation of the EMT program by loss of E-cadherin, mediated by upregulated EMT-TF Snail, Twist, or Zeb1, in breast cancer cells, induces Hedgehog signaling in the pathway involved in stemness induction [13,14].

The tumor suppressor p53 is known to play a key role in preventing tumor development [15]. p53 can inhibit EMT through a miRNA-dependent mechanism that targets key EMT regulators such as Zeb1, Zeb2, Snail, Slug, and Twist1, as well as suppress its associated stem cell phenotype in different types of cancers [16,17]. The wealth of literature data strongly suggests that the p53 activity and protein stability depend on its interactions with other proteins, including the dynamic interplay between E3 ubiquitin ligases/deubiquitinases [18,19]. Furthermore, genotoxic and other forms of stress induce specific post-translational modifications of p53 thereby affecting its interactome [20]. Of note, proteasomes themselves also undergo post-translational changes in response to DNA damage bringing another level of complexity to the regulatory network of p53 interactions [21,22]. It is plausible that Zeb1 and p53 can mutually affect each other’s function on the protein level, which warrants an investigation of their protein interactions. Another important feature of tumors is their heterogeneity mediated by the presence of so-called cancer stem cells (CSCs). It is well known that p53 controls the processes of self-renewal and differentiation of stem cells and cancer stem cells in particular [16]. Subsequently, disruption of the interaction of p53 with its principle E3 ubiquitin ligase MDM2 activates p53 signaling and reduces the MEC CSC population [23]. According to recent studies, loss of or overexpression of mutant p53 in SCC cells leads to increased expression of type 2 Deiodinase (D2), which is associated with a low survival rate in patients with various types of tumors [24].

In a previous work [25], we studied the interactome of the Zeb1 transcription factor in MCF-7/Zeb1 breast carcinoma cells. In the present work, we extended our studies on the possible effects of p53 on the Zeb1 interactome in the sub-population of Zeb1-expressing MCF-7 breast cancer cells enriched for stem cell markers. Our results suggest that the p53 protein differentially affects the interactome of Zeb1 isolated from breast cancer stem cells (BCSCs) versus regular MCF-7 cells. Accordingly, we hypothesize that certain components of Zeb1 interactome specific for BCSCs may serve as novel therapeutic targets for treatment of breast cancer metastasis.

## 2. Results

### 2.1. Cell Model Validation

**Figure 1:**
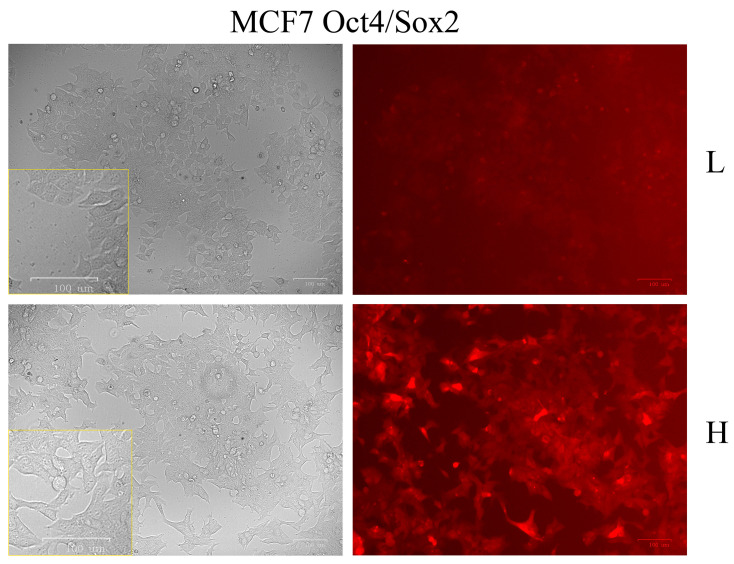
Different expression levels of stemness factors Oct4 and Sox2 in MCF-7/Zeb1 Oct4/Sox2 cells according to the activity of SORE6-mCherry reporter plasmid under light (**left** panel) and fluorescence microscopy (**right** panel): L, H, denote low and high expression levels of Oct4/Sox2 stemness factors, respectively.

Since EMT was reported to be associated with cancer stem cell features [26] and Zeb1 was implicated in this process [27], we decided to investigate the protein composition of Zeb1 interactome in breast cancer stem cells. To this end, we constructed a fluorescence protein coding plasmid (SORE6-mCherry), in which the expression of mCherry protein was driven by Oct4/Sox2 response elements. MCF7/Zeb1 cells were transduced with this lentiviral vector to select for the population of cancer cells enriched in Oct4/Sox2 stem factors. For subsequent comparison of the cancer stem cell population with isogenic cells, MCF-7 clones bearing the genome-integrated SORE6-mCherry construct were divided into two groups according to the level of Oct4/Sox2 activity (as indirectly judged by the intensity of mCherry fluorescence): low (L) and high (H) (Figure 1). Of note, these two populations of cells differed from each other not only by the intensity of fluorescence but also by their phenotypes and proliferation rates. 

**Figure 2:**
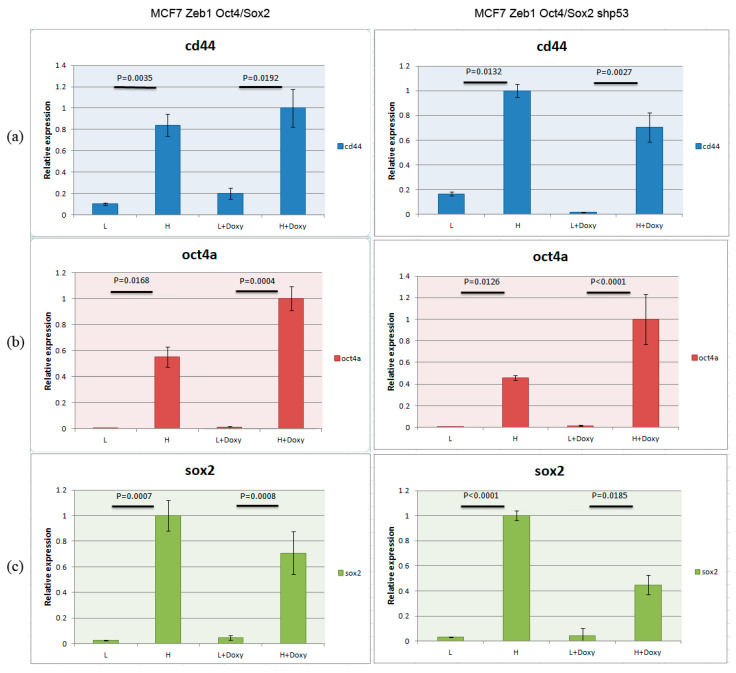
MCF-7-Zeb1 cells with high expression of mCherry exhibit traits of cancer stem cells. RT-PCR with oligonucleotides specific to stemness factors ((**a**): CD44, (**b**): Oct4, (**c**): Sox2) were performed on MCF-7/Zeb1 cells with low (L) and high (H) expression of the SORE6-mCherry reporter. Cells were non-treated or treated with doxycycline to induce the expression of Zeb1. These cells (wt p53, **left**) were treated identically to the cells with knocked down expression of p53 (shRNA-p53, **right**).

To further characterize stemness of these breast cancer cells, we induced the expression of Zeb1 by doxycycline in these two populations of cells (low and high mCherry) (Figure 2, left panels). The non-induced and induced cells were compared for their expression of stemness factors (CD44, Oct4, Sox2) (Figure 2a–c left panels, respectively) by RT-PCR. As evident from the results shown in Figure 2, mCherry Oct4/Sox2-expressing cells (H) were enriched with markers specific for breast cancer stem cells (CD44, Oct4a, and Sox2) compared to isogenic cells with low fluorescence of mCherry (L). Furthermore, the presence of Zeb1 in these cells further enhanced the expression of stem cell markers except for Sox2 expression (which decreased after Zeb1 induction compared to the non-induced cells). 

**Figure 3:**
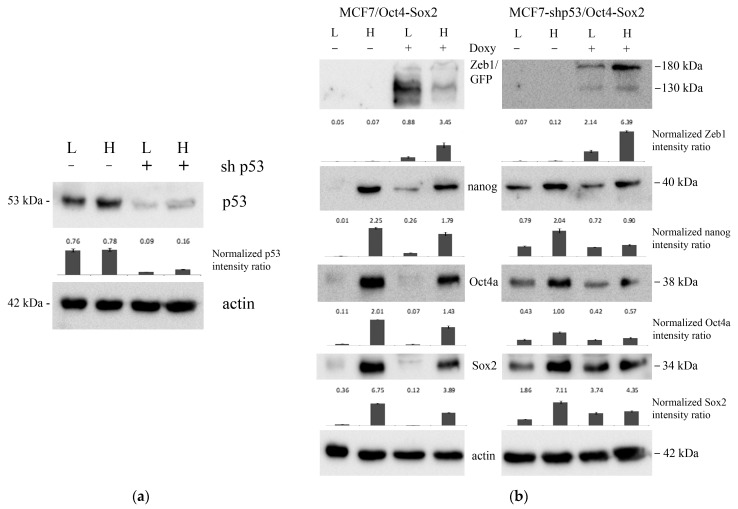
Western blot analysis of MCF-7/Zeb1 cells with different status of p53 (wt and shp53) divided into two groups according to low (L) or high (H) expression of the SORE6-mCherry reporter. (**a**) Immunoblots of total lysates prepared from MCF-7/Zeb1 cells (p53+ or p53-) stained for p53. Actin was used as the loading control. (**b**) Immunoblots of MCF-7/Zeb1 Oct4/Sox2 cells: wild-type p53 (**left** panel), shp53 (**right** panel) stained with Zeb1, Nanog, Oct4 and Sox2 antibodies, respectively. The actin signal was used as the loading control. L, H, − denote low and high level Oct4/Sox2-expressing clones, respectively.

To assess the effect of p53 on stemness of breast cancer cells we repeated the procedure of cancer stem cell enrichment in MCF-Zeb1 cells with either knocked down or normal expression of p53 (Figure 2, right panels). Surprisingly, we did not see a dramatic effect of p53 on the expression of stem cell markers as judged by RT-PCR. In parallel, we confirmed the efficacy of induction of Zeb1 by Western blotting. We also validated the efficiency of p53 knockdown by immunoblotting with appropriate antibodies (Figure 3a) and assessed the changes in the protein expression Nanog stemness factor (Figure 3b).

### 2.2. Co-Immunoprecipitation and Mass Spectrometric Identification of Zeb1 EMT-TF Interacting Proteins

Following the immunoprecipitation of Zeb1-interacting proteins from the engineered MCF7 SORE6-mCherry cell lines described above, the identity of these proteins was revealed by LC-MALDI TOF/TOF spectrometry. In 8 samples (4 for each cell model), about 800 proteins were reliably identified. A significant part of these proteins resulted from nonspecific binding to single-chain antibodies from llama against the green fluorescent protein (GFP) immobilized on magnetic beads. However, over 60 proteins were found to specifically interact with Zeb1 as they appeared only in samples after Zeb1 induction, of which about a quarter were common for both p53+ and p53- variants. Importantly, 36 interactors were found in the cells with normal expression of p53, and 15 were present only in the cells with knockdown of p53. The results of the mass spectrometric analysis are presented in Table 1, Table S1 and Table S2.

**Figure 4:**
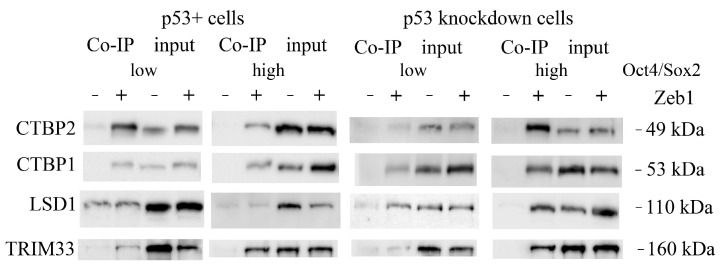
Western blot analysis of Zeb1 interactome components co-immunoprecipitated from MCF-7/Zeb1 cells with different status of p53 (wt and shp53) divided into two groups according to low or high expression of the SORE6-mCherry reporter.

We confirmed the selected interactants (MGMT, ELAVL1 (HuR), CTBP1/2, LSD1 (KDM1A) and TRIM33 (TIF1γ)) by immunoblotting (Figure 4), which allowed us to assess the changes in the content of studied proteins from the selected clones.

**Figure 5:**
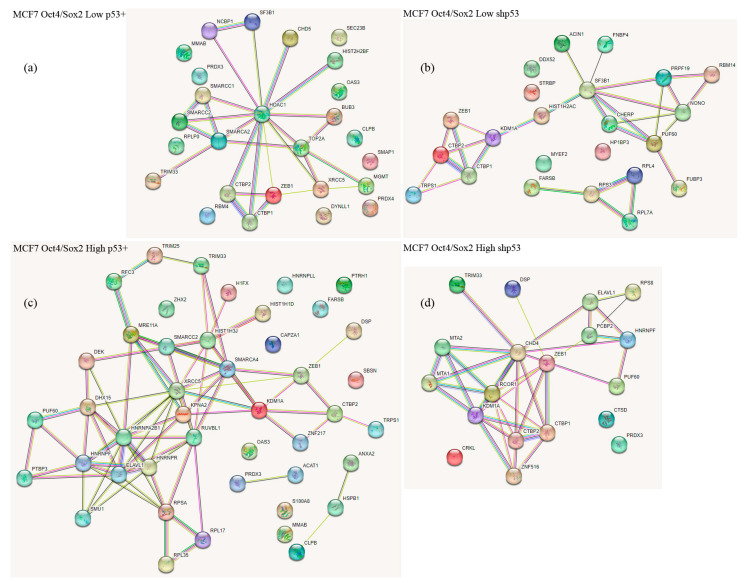
STRING database analysis of protein networks, showing proteins connected to the Zeb1 node. Zeb1 interactomes of MCF-7/Zeb1 cells with different status of p53 (wt and shp53) divided into two groups according to low (L) or high (H) expression of the SORE6-mCherry reporter. (**a**): MCF-7/Zeb1 wt-p53 cell clones with low Oct4/Sox2 expression levels; (**b**): MCF-7/Zeb1 wt-p53 cell clones with high Oct4/Sox2 expression levels; (**c**): MCF-7/Zeb1 shp53 cell clones with low Oct4/Sox2 expression levels; (**d**): MCF-7/Zeb1 shp53 cell clones with high Oct4/Sox2 expression levels.

The identified Zeb1-bound proteins could be attributed to several functional groups as judged from the bioinformatics analysis using the STRING database (https://string-db.org (accessed on 3 February 2023)). We also noticed that the presence of p53 strongly attenuated the binding of both CTBP1 and CTBP2 to Zeb1 (Table 1) irrespective of the cell stemness status. In addition to those interactors, LSD1 lysine demethylase appeared to specifically interact with Zeb1 only in the cells with attenuated expression of p53 (p53KD clones). Furthermore, the REST corepressor was also detected in the p53KD cells but only in the “high” Oct4/Sox2 clone which correlates with the highest expression of stemness factors (Table S1). On the contrary, the presence of p53 favored the interaction of Zeb1 with such proteins as XRCC5 (DNA repair) and SMRCA2 (chromatin remodeling). In this respect, it should be noted that Zeb1 was previously shown to interact with the SWI/SNF chromatin remodeling complex (BAF) [28]. Desmoplakin (a critical part of desmosomes), heterogeneous nuclear ribonucleoprotein F (the mRNA splicing factor), and ELAVL1 (the mRNA binding protein) were found to be associated with Zeb1 only in the “high” population of cells which strongly expressed Oct4/Sox2 (Figure 5). The latter observation, taken together with the fact that another mRNA splicing factor, SF3B1, also binds Zeb1 specifically in non-stem cancer cells, suggests that the composition of Zeb1-specific splicing factors may be an important regulatory mechanism for cancer stemness. 

## 3. Discussion

Most tumors of various origin contain fractions of cancer stem cells that provide tumors with high levels of plasticity [29]. The Zeb1 transcription factor plays an important role in the processes of dynamic interconversion of tumor stem and non-stem cells. To better understand the role of Zeb1 in this process, we decided to analyze the interactomes of Zeb1 isolated from control and cancer-stem cell-like cultures.

It should be noted that most of the studies on Zeb1-interacting proteins have been performed on HEK293 cells derived from the human embryonic kidney [30]. These cells do not undergo EMT even on forced expression of Zeb1, hence questioning the biological significance of the obtained results. In contrast, in our MCF-7-based breast cancer cell model, the ectopic expression of Zeb1 confers a partially mesenchymal phenotype. The latter highlights the importance of Zeb1 interactions observed in these cells. 

Comparison of our previous proteomic results [25] with the extremely scarce literature data related to proteins interacting with Zeb1 [30,31], prompted us to conclude that the p53 tumor suppressor status affects the composition of Zeb1 interactome. In the present paper, we found that the interactome of Zeb1 includes components of the SWI/SNF chromatin remodeling complex, NuRD, and LSD1-CtBP-CoREST transcription corepressor complexes, as well as several proteins interacting with the latter. The composition of the Zeb1 interactome shows dependence on the status of p53 and the expression levels of stemness factors (Oct4, Sox2, Nanog).

At present, we do not know whether p53 affects the interactome of Zeb1 at the level of direct protein-protein interactions with Zeb1-associated proteins or indirectly, via the “regulation of regulators” thereby affecting the availability of Zeb1-associated proteins. Future studies should address this interesting question. On the related note, it is well known that the interactome of p53 can be modified pharmacologically. For example, p53 interacts with Mdm2 and this interaction can be abolished by several small molecules, including Nutlin-3A [32,33,34,35,36]. It would be interesting to see whether nutlin-3A and other experimental molecules that affect p53 interactions can also affect the interactome of Zeb1.

Analysis of the literature on Zeb1-dependent transcriptional regulation suggests that ZEB1 requires an interaction with the CtBP-LSD1-CoREST complex to repress transcription of its target genes [37]. In line with this, we found that all samples of Zeb1-bound proteins contained the components of the LSD1-CTBP corepressor complex, yet varied in the content of other proteins that interacted with Zeb1. 

Two classes of chromatin modifiers are commonly associated with CtBP: histone deacetylases HDAC1 and HDAC2 and the H3K4Me2 demethylase, LSD1 [38,39,40]. LSD1 (KDM1A) is a monoamine oxidase that catalyzes the progressive removal of H3K4Me2 and H3K4Me1 activation marks by demethylation, thereby attenuating the appearance of the H3K4Me3 activating modification, which plays a key role in EMT modulation [41].

The presence of histone lysine demethylase, LSD1, a component of the CoREST-CtBP corepressor complex [37], was found to be elevated in the Zeb1 interactome of p53KD “HIGH” Oct4/Sox2 cells. 

The Zeb1 interactome (mainly isolated from the wt-p53-expressing cells) contains components of the ATP-dependent chromatin-remodeling complex BAF (SWI/SNF), which facilitates the access of transcription factors to chromatin [42]. In this respect, we detected six SWI/SNF chromatin-remodeling proteins: SMARCC1, SMARCC2, SMARCA2 (SNF2L2), SMARCA4 (BRG1), as well as CHD4 and CHD5 (which belong to the chromodomain helicase DNA-binding protein family) as interactors of Zeb1. The CHD family of proteins consists of nine members divided into three sub-families. CHD3-CHD5 proteins belong to one sub-family [43]. The canonical components of this complex are HDAC1 and HDAC2; MTA1 and MTA2; RBBP4 and RBBP7; GATA2DA and GATA2DB; and MDB2 and MDB3. Kolla and co-authors [43] described a hypothetical CHD5-NuRD complex which, they suggest, is identical to the CHD4-NuRD complex. 

As mentioned above, among Zeb1 interactors we found several components of the ATP-dependent nucleosome remodeling and deacetylase (NuRD) complex: CHD4, CHD5, HDAC1, MTA1, MTA2. In the “high” Oct4/Sox2 shp53 clone we identified a chromodomain helicase DNA-binding protein 4 (CHD4) as an interacting partner of Zeb1. The former is known to be the key catalytic sub-unit of the NuRD complex. CHD4/NuRD plays a critical role in regulation of gene expression via the modulation of chromatin structure and assembly [44]. CHD4 has been well documented to be involved in gene repression in cancer cells. Our data revealed two additional components of the NuRD complex (metastasis-associated proteins, MTA1 and MTA2) in the shp53 HIGH clone.

A significant number of Zeb1 interactors belongs to the group of RNA-binding proteins that have emerged recently as important post-transcriptional modulators of carcinogenesis, but only a few have had their roles in breast cancer confirmed. One of the Zeb1 interactors is the splicing factor SF3B1, which is overexpressed in breast cancer tissue compared to normal tissue. SF3B1, whose overexpression is associated with lymph node metastasis [45], was found preferentially in “low” Oct4/Sox2 clones irrespective of the p53 status. Another RNA binding protein, ELAVL1 (HuR), was detected, on the contrary, in “high” Oct4/Sox2 clones only. The HuR protein was recently shown to be a novel target of p53-specific Pirh2 E3 ubiquitin ligase [46]. We have previously reported that ELAVL1 selectively augmented the expression of another oncogene, c-fos [47]. Our results that Zeb1 interacts with RNA-binding proteins are in line with the notion that aberrant expression of ZEB1 is involved in the regulation of epithelial splicing regulatory proteins (ESRPs) [48]. All of the above evidence taken together suggest that HuR may be considered as a potential therapeutic target in breast cancer and other tumors.

TRIM25 found in p53+ HIGH clone is known to be estrogen-responsive in BC. TRIM25 interferes with the formation and activity of the ternary complex p53-MDM2-p300, blocking both the polyubiquitination and acetylation of 53. This leads to an increase of the p53 level which, however, is not active, since acetylation is necessary for the transactivation of target genes that inhibit growth and proapoptotic genes. It is likely that the TRIM25 activity can modulate the p53-dependent DNA damage response [49]. Furthermore, TRIM25 can down-regulate p53 activity via the interaction with G3BP2 together with RanBP2, an E3 ligase responsible for sumoylation, SUMO-conjugation of p53, and its androgen-mediated nuclear export [50]. Another tripartite-motif E3 ligase, TRIM33, involved in EMT, has been identified in three MCF7/Zeb1 Oct4/Sox2 clones (except shp53 “low”) [51]. Importantly, TRIM33, as a part of the tripartite complex (TRIM24-TRIM33-TRIM28) can also target p53 for degradation [52]. In general, the TRIM family proteins, via their ubiquitin-dependent targeting of proteins to proteasomes, participate in various cellular processes, including gene regulation [53]. Interestingly, proteasomes themselves can also actively regulate transcription [22].

Collectively, in this study we have identified the interactome of Zeb1 in relevant physiological conditions of EMT and compared the impact of p53 and Oct4/Sox2 stem factors on the composition of Zeb1. Future studies should assess the physiological importance of our discovery.

## 4. Materials and Methods

### 4.1. Cell Culture

To induce the expression of the *Zeb1* gene, we used human breast adenocarcinoma MCF-7 cells of luminal subtype [54] with the rTTA vector integrated into the genome. The *Zeb1* gene fused with the GFP sequence was expressed from the pVI vector (Clontech) under the tetracycline-activated promoter. The cells were cultured according to a standard protocol in a DMEM medium with 10% fetal bovine serum, 0.06% L-glutamine, 0.001% insulin, and 0.004% gentamycin in an incubator in an atmosphere of 5% CO_2_ at 37 °C.

To induce the expression of the Zeb1/GFP fusion protein, doxycycline at a final concentration of 0.5 μg/mL was added to the culture medium for 72 h. Expression of Zeb1 was detected by GFP luminescence using a Zoe fluorescence microscope (BioRad, Hercules, CA, USA).

Cells were transfected by the calcium phosphate method to introduce the SORE6x-dsmCherry-Blast plasmid reporter construct (courtesy of Dr. Tang) based on the lentiviral integrating vector, as well as auxiliary plasmids to provide viral particle according to the standard protocol (https://www.epfl.ch/labs/tronolab/wp-content/uploads/2019/06/LV_production.pdf (accessed on 12 April 2020)). This plasmid contained the Blasticidin resistance gene and the mCherry fluorescent protein expressed under the minimal CMV promoter and the Oct4/Sox2 responsive element (SORE), taken from SORE6x [55] into the lenti-Cas9-Blast plasmid by replacing Cas9 insert with a hexa-repeated SORE sequence (428 bp) under a minimal CMV promoter, using NheI and BamHI sites. Transduced MCF7-Zeb1 and MCF7-Zeb1-shp53 cells were seeded at low density for selection of resistant clones in the blasticidin-containing medium (within 12 days, changing the medium every 2 days). Resistant clones (about 10–30 cells per clone) had a different intensity of mCherry fluorescence. We concluded that clones with different levels of mCherry expression (low (L) and high (H)) also have different levels of the stemness factors expression.

### 4.2. Co-Immunoprecipitation

To isolate the ZEB1 interactome, the MCF7 nuclear extracts were co-immunoprecipitated with llama nano-antibodies against the green fluorescent protein immobilized on magnetic beads (Abcam ab193983). The protein conjugates immunoprecipitated on magnetic beads were washed 4 times with RIPA buffer, then dissolved in the Laemmli buffer and subjected to SDS-PAGE to remove PEG derivatives present in the wash buffer.

### 4.3. Sample Preparation and LC-MALDI Mass Spectrometry

The protein samples were in-gel digested overnight by Trypsin Gold (Promega) followed by extraction and drying in the Martin Christ RVC-2-33IR rotary vacuum concentrator (Martin Christ, Osterode am Harz, Germany). Prior to reversed-phase fractionation, digested samples were resuspended in 50 μL of 1% (*v/v*) formic acid in water and filtered through 0.2 μm PVDF filter. Peptides were separated with a Chromolith CapRod RP-18e HR reversed-phase column (0.1 mm × 150 mm, Merck, Darmstadt, Germany) on a nano LC system (Eksigent NanoLC Ultra 2D+ system, SCIEX, Darmstadt, Germany). A total peptide amount of 600 ng was loaded and separated using a linear gradient of 0–50% B over 115 min followed by 50–100% B for 1 min and 100–100% B for 4 min at a flow rate of 400 nL/min. The mobile phases used were A, 5% acetonitrile with 0.2% (*v/v*) TFA in water and B, 60% (*v*/*v*) acetonitrile in water. The column was operated at a room temperature of 22–24 °C. The effluent from the column was mixed with matrix solution (CHCA 5 mg/mL, 0.2% (*v/v*) TFA in 95% methanol) containing two calibration standards bradykinin 2-9 (30 pM/mL) and ACTH 18-39 (60 pM/mL), at a flow rate of 1.4 μL/min. A micro-fraction collector was used to deposit 1 mm spots every 5 s, and a total of 1408 fractions were collected in a 44 × 32 array for each nano LC run. The column was washed with a gradient (0–100–100% B for 5 min and 2 min, respectively, at a flow rate of 800 nL/min) and equilibrated to 0% B for 3.5 min before subsequent injections.

We analyzed the fractionated samples with a TOF/TOF 5800 System (SCIEX, Darmstadt, Germany) instrument operated in the positive ion mode. We set the MALDI stage to continuous motion mode. MS data was acquired at 2800 laser intensity with 1000 laser shots/spectrum (200 laser shots/sub-spectrum) and MS/MS data were acquired laser intensity at 3600 with a DynamicExit algorithm and a high spectral quality threshold or a maximum of 1000 laser shots/spectrum (250 laser shots/sub-spectrum). Up to 25 top precursors with S/N > 40 in the mass range 750–4000 Da were selected from each spot for MS/MS analysis.

We used the Protein Pilot 5.0 software (SCIEX, Darmstadt, Germany) with the Paragon algorithm 5.0 in thorough mode, for the MS/MS spectra search against the Uniprot human database. Carbamidomethyl cysteine was set as a fixed modification. False discovery rate (FDR) analysis was done by analysis of reversed sequences using the embedded PSEP tool. The MS/MS data was converted to mzidentml format for further analysis using Scaffold 4.0 software. The mass spectrometry proteomics data have been deposited at the Mendeley Data, V1, doi: 10.17632/xpzrjyf935.1.

### 4.4. Western-Blotting

After separation in SDS-PAGE (AA: 10%, AA/BisAA ratio: 36:1), the proteins were transferred onto PVDF membranes following overnight incubation with specific primary antibodies. The following primary antibodies were used: anti-actin (Sigma A5441, Sigma-Aldrich, St. Louis, MO, USA), anti-Zeb1 (Sigma AMAb90510, Sigma-Aldrich, St. Louis, MO, USA), anti-Nanog (Santa Cruz sc-293121, Dallas, TX, USA), anti-Oct4 (Cell Signaling #2890s, Danvers, MA, USA), anti-Sox2 (Cell Signaling #3579s, Danvers, MA, USA), anti-CTBP2 (Abcam ab128871, Cambridge, UK), anti-CTBP1 (Sigma HPA018987, Sigma-Aldrich, St. Louis, MO, USA), anti-LSD1 (Sigma ABE365, Sigma-Aldrich, St. Louis, MO, USA), anti-TRIM33 (Sigma HPA004345, Sigma-Aldrich, St. Louis, MO, USA), and anti-p53 (Cell Signaling #46565, Danvers, MA, USA). The secondary antibodies were from Sigma: anti-mouse (A9917) and anti-rabbit (A0545). Bound antibodies were visualized using the SuperSignal West Femto Maximum Sensitivity Substrate ECL kit (Thermo scientific, Waltham, MA, USA), chemiluminescence was detected using ChemiDoc (BioRad, Hercules, CA, USA).

### 4.5. Real-Time Polymerase Chain Reaction

Total RNA was isolated from cells using a TRI Reagent (Sigma-Aldrich, St. Louis, MO, USA). cDNA was then synthesized by reverse transcription using MMLV RT kit (Eurogen, Moscow, Russia). qPCR mix-HS SYBR master mix (Eurogen, Moscow, Russia) was used for real-time PCR, with GAPDH as the internal control. The primers are listed in Table 2. The total qPCR reaction volume was 20 μL and consisted of 2 μL of cDNA, 0.1 μL of each primer, 4 μL of qPCR mix, and 13.8 μL of ddH_2_O. The PCR reaction program was as follows: 95 °C for 1 min; 40 cycles of 95 °C for 15 s, 60 °C for 15 s, then heated to 95 °C for 60 s; and cooled to 4 °C for 5 min. To estimate the relative gene expression the ΔΔCt method was used.

### 4.6. Statistical Analysis

Data are presented as mean with standard deviation (SD) or standard error of the mean (SEM) and as median with quartiles when the experiment is repeated at least three times. Statistical significance was analyzed using Student’s *t*-test or Mann–Whitney test.

## 5. Conclusions

Using a novel fluorescent reporter construction and small hairpin RNA we obtained CSC-like cell clones differing in both Oct4/Sox2 and p53 expression. We have shown the Zeb1 interactome from those clones to contain the components of two complexes: BAF chromatin remodeling and LSD1-CtBP-CoREST corepressor. We also found the composition of Zeb1 interactome to depend on both the p53 status and the Oct4/Sox2 expression level which suggests the specificity of Zeb1 interactions to be likely affected by stemness. Current data presented here provide an opportunity for further studies of Zeb recruitment in driving cancer stemness and metastasis.

## Figures and Tables

**Table 1:** Zeb1-interacting proteins identified by LC-MALDI TOF/TOF.

Proteins Names	Number of Confident Peptides in MCF-7 Clones			
	LOW OS-mC p53+cells (L-P53WT)	HIGH OS-mC p53+cells (H-P53WT)	LOW OS-mC shp53 Cells (L-shP53)	HIGH OS-mC shp53 Cells (H-shP53)
C-terminal-binding protein 2 (CTBP2)	7	5	12	14
2′-5′-oligoadenylatesynthase 3 (OAS3)	6	2	0	0
Caseinolytic peptidase B protein homolog (CLPB)	4	3	0	0
Corrinoid adenosyltransferase (MMAB)	2	2	0	0
SWI/SNF complex sub-unit SMARCC2 (SMARCC2)	6	6	0	0
X-ray repair cross-complementing protein 5 (XRCC5)	5	3	0	0
Thioredoxin-dependent peroxide reductase, mitochondrial (PRDX3)	6	5	0	4
E3 ubiquitin-protein ligase TRIM33 (TRIM33)	6	4	0	11
C-terminal-binding protein 1 (CTBP1)	6	0	11	10
Splicing factor 3B sub-unit 1 (SF3B1)	3	0	3	0
Methylated-DNA--protein-cysteine methyltransferase (MGMT)	5	0	0	3
Heterogeneous nuclear ribonucleoprotein F (HNRNPF)	0	9	0	4
Poly(U)-binding-splicing factor PUF60 (PUF60)	0	2	4	4
Zinc finger transcription factor Trps1 (TRPS1)	0	3	3	0
Phenylalanine--tRNA ligase beta sub-unit (FARSB)	0	4	4	0
Desmoplakin (DSP)	0	3	0	4
ELAV-like protein 1 (ELAVL1)	0	5	0	4
Lysine-specific histone demethylase 1A (KDM1A)	0	0	8	9

FDR = 1%, all MS/MS experiments were at least triplicated. NP—number of confident peptides (*p* < 0.01). Interactomes of p53+ or p53- (shP53) MCF-7 cells with induced expression of Zeb1 expressing either low (L) or high levels (H) of Oct4/Sox2 factors (based on the activity of the Oct4/Sox2 mCherry (OS-mC) reporter) are presented in the Table 1.

**Table 2:** Oligonucleotide sequences used in qPCR.

Gene	5′-3′ Primer Sequence
*Oct4a*	F: CTTCTGCTGATCCTGTCTGATG
	R: TGCTGTGAAGGGAGATGTATTG
*Cd44*	F: GTCTCCTCTGACTTCAACAGCG
	R: ACCACCCTGTTGCTGTAGCCAA
*Sox2*	F: GGCATACACCTACTCAACTACGG
	R: TGGGCGGTGTAGAATCAGAGTC

## Data Availability

The mass spectrometry proteomics data have been deposited in Mendeley Data, V1, 26 April 2023, https://data.mendeley.com/datasets/xpzrjyf935/1.

## Supplementary Materials

The following supporting information can be downloaded at: https://www.mdpi.com/article/10.3390/ijms24129806/s1.

## Abbreviations

BCSCs	breast cancer stem cells
CHCA	α-cyanohydroxycinnamic acid
CMV	cytomegalovirus
CSCs	cancer stem cells
EMT	epithelial-to-mesenchymal transition
EMT-TF (TF-EMT)	transcription factors that govern EMT
ESRPs	epithelial splicing regulatory proteins
FDR	false discovery rate
GFP	green fluorescent protein
LC-MALDI TOF/TOF	liquid chromatography/matrix-assisted laser desorption/ionization time-of-flight tandem mass spectrometry
OS-mC	Oct4/Sox2 mCherry reporter
SDS-PAGE	sodium dodecylsulphate polyacrylamide gel electrophoresis
SORE	Oct4/Sox2 responsive element
