# Supplementary material for: p53 Affects Zeb1 Interactome of Breast Cancer Stem Cells
Source: Int J Mol Sci. 2023 Jun 6;24(12):9806. doi: 10.3390/ijms24129806 (PMC10297930; doi:10.3390/ijms24129806)
Supplement: Supplementary file 1 [file ijms-24-09806-s001.zip › ijms-2398387-supplementary.pdf]

Supplementary tables

Table S1. The unique proteins for each clone identified by LC-MALDI TOF/TOF mass spectrometry

MCF7/Zeb1 OS-mC LOW p53+ cells (L-P53WT)		MCF7/Zeb1 OS-mC HIGH p53+ cells (H-P53WT)		MCF7/Zeb1 OS-mC LOW shp53 cells (L-shP53)		MCF7/Zeb1 OS-mC HIGH shp53 cells (H-shP53)	
Protein name	NP	Protein name	NP	Protein name	NP	Protein name	NP
60S acidic ribosomal protein P0 **(RPLP0)**	3	40S ribosomal protein SA **(RPSA)**	3	40S ribosomal protein S3 **(RPS3)**	5	40S ribosomal protein S8 **(RPS8)**	3
Chromodomain-helicase-DNA-binding protein 5 **(CHD5)**	4	60S ribosomal protein L23 **(RPL23)**	3	60S ribosomal protein L4 **(RPL4)**	4	Cathepsin D **(CTSD)**	3
DNA topoisomerase 2-alpha **(TOP2A)**	6	60S ribosomal protein L35 **(RPL35)**	3	60S ribosomal protein L7a **(RPL7A)**	4	Chromodomain-helicase-DNA-binding protein 4 **(CHD4)**	9
Dynein light chain 1, cytoplasmic **(DYNLL1)**	3	Acetyl-CoA acetyltransferase, mitochondrial **(ACAT1)**	9	Apoptotic chromatin condensation inducer in the nucleus **(ACIN1)**	7	Crk-like protein **(CRKL)**	3
Histone deacetylase 1 **(HDAC1)**	5	Annexin A2 **(ANXA2)**	9	Calcium homeostasis endoplasmic reticulum protein **(CHERP)**	4	Metastasis-associated protein MTA1 **(MTA1)**	4
Histone H2B type 2-F **(HIST2H2BF)**	3	Double-strand break repair protein MRE11 **(MRE11)**	4	Far upstream element-binding protein 3 **(FUBP3)**	8	Metastasis-associated protein MTA2 **(MTA2)**	4
Mitotic checkpoint protein BUB3 **(BUB3)**	3	E3 ubiquitin/ISG15 ligase TRIM25 **(TRIM25)**	3	Formin-binding protein 4 **(FNBP4)**	3	Poly(rC)-binding protein 2 **(PCBP2)**	3
Nuclear cap-binding protein subunit 1 **(NCBP1)**	3	F-actin-capping protein subunit alpha-1 **(CAPZA1)**	4	Heterochromatin protein 1-binding protein 3 **(HP1BP3)**	3	REST corepressor 1 **(RCOR1)**	3
Peroxiredoxin-4 **(PRDX4)**	4	Heat shock protein beta-1 **(HSPB1)**	3	Histone H2A type 1-C **(HIST1H2AC)**	3	Zinc finger protein 516 **(ZNF516)**	3
Probable global transcription activator SNF2L2 **(SMARCA2)**	5	Heterogeneous nuclear ribonucleoproteins A2/B1 **(HNRNPA2B1)**	3	Myelin expression factor 2 **(MYEF2)**	6		
Protein transport protein Sec23B **(SEC23B)**	4	Heterogeneous nuclear ribonucleoprotein L-like **(HNRNPLL)**	3	Non-POU domain-containing octamer-binding protein **(NONO)**	4		
RNA-binding protein 4 **(RBM4)**	3	Heterogeneous nuclear ribonucleoprotein R **(HNRNPR)**	5	Pre-mRNA-processing factor 19 **(PRPF19)**	8		
Stromal membrane-associated protein 1 **(SMAP1)**	4	Histone H1.3 **(HIST1H1D)**	8	Probable ATP-dependent RNA helicase DDX52 **(DDX52)**	3		
SWI/SNF complex subunit SMARCC1 **(SMARCC1)**	4	Histone H1x **(H1FX)**	4	Regulation of nuclear pre-mRNA domain-containing protein 2 **(RPRD2)**	6		
		Histone H3.1 **(HIST1H3J)**	3	RNA-binding protein 14 **(RBM14)**	8		

		Importin subunit alpha-1 **(KPNA2)**	3	Spermatid perinuclear RNA-binding protein **(STRBP)**	4		
		Polypyrimidine tract-binding protein 3 **(PTBP3)**	4				
		Pre-mRNA-splicing factor ATP-dependent RNA helicase DHX15 **(DHX15)**	3				
		Probable peptidyl-tRNA hydrolase **(PTRH1)**	4				
		Protein DEK **(DEK)**	3				
		Protein S100-A8 **(S100A8)**	3				
		Replication factor C subunit 3 **(RFC3)**	3				
		RuvB-like 1 **(RUVBL1)**	2				
		Suprabasin **(SBSN)**	3				
		Transcription activator BRG1 **(SMARCA4)**	4				
		WD40 repeat-containing protein SMU1 **(SMU1)**	4				
		Zinc finger protein 217 (**ZNF217)**	2				
		Zinc fingers and homeoboxes protein 2 (**ZHX2)**	4				

Table S2. Non-specifically bound proteins (at least nine biological repeats for each column)

Protein name	Number of peptides	
	p53+cells	shp53 cells
3-hydroxyacyl-CoA dehydrogenase type-2 (**HSD17B10**)	**7**	**0**
40S ribosomal protein S20 **(RPS20)**	**5**	**0**
60S ribosomal protein L23 **(RPL23)**	**0**	**5**
Actin, cytoplasmic 1 **(ACTB)**	**9**	**0**
Actin, cytoplasmic 2 **(ACTG1)**	**0**	**7**
Aspartyl aminopeptidase **(DNPEP)**	**5**	**0**
ATP-dependent RNA helicase A **(DHX9)**	**8**	**15**
ATP-dependent RNA helicase DDX3X **(DDX3X)**	**9**	**11**
DNA topoisomerase 1 **(TOP1)**	**7**	**17**
DNA topoisomerase 2-beta **(TOP2B)**	**11**	**0**
DNA-3-methyladenine glycosylase **(MPG)**	**6**	**10**
DNA-dependent protein kinase catalytic subunit **(PRKDC)**	**35**	**48**
Double-stranded RNA-specific adenosine deaminase **(ADAR)**	**9**	**16**
Elongation factor 1-alpha 1 **(EEF1A1)**	**0**	**14**
Far upstream element-binding protein 2 **(KHSRP)**	**14**	**15**
Far upstream element-binding protein 3 **(FUBP3)**	**7**	**0**

General transcription factor II-I **(GTF2I)**	**8**	**12**
Heat shock 70 kDa protein 1A **(HSPA1A)**	**7**	**0**
Heat shock cognate 71 kDa protein **(HSPA8)**	**11**	**19**
Heterogeneous nuclear ribonucleoprotein H **(HNRNPH1)**	**8**	**16**
Heterogeneous nuclear ribonucleoprotein K **(HNRNPK)**	**14**	**13**
Heterogeneous nuclear ribonucleoprotein L **(HNRNPL)**	**0**	**12**
Heterogeneous nuclear ribonucleoprotein M **(HNRNPM)**	**13**	**29**
Histone H1.2 **(HIST1H1C)**	**7**	**0**
Histone H4 **(HIST4H4)**	**5**	**0**
Kinesin-like protein KIF22 **(KIF22)**	**6**	**0**
Lamina-associated polypeptide 2, isoform alpha **(TMPO)**	**8**	**0**
Matrin-3 **(MATR3)**	**15**	**18**
Methylcrotonoyl-CoA carboxylase beta chain, mitochondrial **(MCCC2)**	**11**	**0**
Nuclear mitotic apparatus protein 1 **(NUMA1)**	**45**	**69**
Nucleophosmin **(NPM1)**	**0**	**10**
Peptidyl-prolyl cis-trans isomerase A **(PPIA)**	**0**	**9**
Peroxiredoxin-1 **(PRDX1)**	**13**	**16**
Poly [ADP-ribose] polymerase 1 **(PARP1)**	**7**	**0**
Polypyrimidine tract-binding protein 1 **(PTBP1)**	**9**	**11**
Probable ATP-dependent RNA helicase DDX17 **(DDX17)**	**24**	**18**
Probable ATP-dependent RNA helicase DDX5 **(DDX5)**	**31**	**29**
Protein disulfide-isomerase A3 **(PDIA3)**	**8**	**0**
Protein disulfide-isomerase A6 **(PDIA6)**	**16**	**11**
Protein disulfide-isomerase **(P4HB)**	**8**	**0**
Protein RCC2 **(RCC2)**	**15**	**21**
Putative elongation factor 1-alpha-like 3 **(EEF1A1P5)**	**8**	**0**
RNA-binding protein 25 **(RBM25)**	**0**	**11**
Serine/arginine repetitive matrix protein 2 **(SRRM2)**	**17**	**17**
Splicing factor, proline- and glutamine-rich **(SFPQ)**	**5**	**7**
Stress-70 protein, mitochondrial **(HSPA9)**	**8**	**9**
Thioredoxin domain-containing protein 5 **(TXNDC5)**	**7**	**0**
TAR DNA-binding protein 43 **(TARDBP)**	**0**	**5**
Tubulin alpha-1B chain **(TUBA1B)**	**0**	**16**
Tubulin beta-4B chain **(TUBB4B)**	**5**	**20**
Tudor-interacting repair regulator protein **(NUDT16L1)**	**6**	**8**
X-ray repair cross-complementing protein 6 **(XRCC6)**	**5**	**14**
